# Evaluating effects of recent changes in NHS resource allocation policy on inequalities in amenable mortality in England, 2007–2014: time-series analysis

**DOI:** 10.1136/jech-2018-211141

**Published:** 2018-11-23

**Authors:** Jonny Currie, Maria Guzman Castillo, Victor Adekanmbi, Ben Barr, Martin O’Flaherty

**Affiliations:** 1 Public Health Wales, Cardiff, UK; 2 Department of Public Health, University of Liverpool, Liverpool, UK; 3 Division of Population Medicine, School of Medicine, Cardiff University, Neuadd Meirionnydd, University Hospital of Wales, Cardiff, UK

**Keywords:** inequalities, policy, time-series, public health

## Abstract

**Background:**

Health investment in England post-2010 has increased at lower rates than previously, with proportionally less being allocated to deprived areas. This study seeks to explore the impact of this on inequalities in amenable mortality between local areas.

**Methods:**

We undertook a time-series analysis across 324 lower-tier local authorities in England, evaluating the impact of changes in funding allocations to health commissioners from 2007 to 2014 on spatial inequalities in age-standardised under-75 mortality rates for conditions amenable to healthcare for men and women, adjusting for trends in household income, unemployment and time-trends.

**Results:**

More deprived areas received proportionally more funding between 2007 and 2014, though the reorganisation of commissioning in 2012 stalled this. Funding increases to more deprived local areas accounted for a statistically significant reduction in inequalities in male amenable mortality between local areas of 13 deaths per 100 000 (95% CI 2.5 to 25.9). Funding changes were associated with a reduction in inequalities in female amenable mortality of 7.0 per 100,000, though this finding did not reach significance (p=0.09).

**Conclusion:**

Current National Health Service (NHS) resource allocation policy in England appears to be contributing to a convergence in health outcomes between affluent and deprived areas. However, careful surveillance is needed to evaluate whether diminished allocations to more deprived areas in recent years and reduced NHS investment as a whole is impacting adversely on inequalities between groups.

## Introduction

Since 2010 the National Health Service (NHS) in England has faced significant funding pressures in an apparent effort to reduce deficits following the economic crisis.^1 2^ Following prior record investment,^3^ NHS services received from 2010 to 2016 on average 1.3% additional funding, against demand increases of over 3% annually.^4^ Given rising pressures,^5^ many have questioned the sustainability of such budgetary pressures, given unparalleled scarcity in NHS history.

Wider public services have faced similar or more stringent cuts which appear to be harming population health.^6–9^ One study found areas experiencing higher unemployment rises to see greater numbers of suicides, with unemployment correlated with council budget cuts.^6^ Another study linked cuts to income-support for low-income pensioners with rises in old-age mortality.^7^ Inequalities in mental health problems also widened from 2009 to 2013, mirroring trends in unemployment and wages.^8^ Of particular concern, one study estimated constrained healthcare spending from 2010 to 2014 to be associated with approximately 45 000 excess deaths, predominantly among over-60s and those in care homes^9^; while the precise determinants of these are unclear, recent data released by the Office for National Statistics (ONS), corroborated by Public Health England,^10^ the Kings Fund,^11^ the Institute of Health Equity^12^ and actuarists^13^ have demonstrated since the early 2010s a significant deceleration in the long-term trend of reductions in mortality rates in England and Wales.^14^ More recent data suggest reductions in life expectancy since 2014, in contrast to continuing increases in several other high-income countries.^15^

The preceding period saw a different political programme and improvement in population health. From 2003, the English government set out to narrow inequalities in infant mortality and life expectancy by 10%.^16^ Initial analyses suggested the strategy was ineffective,^17 18^ however subsequent evaluations concluded it achieved its aims.^19^

Evaluating the specific impact of healthcare on health inequalities is challenging, particularly since the drivers of such disparities are predominantly economic and environmental.^20^ One approach is to solely evaluate deaths that would be avoided were timely and quality healthcare available, or ‘amenable mortality’.^21 22^ One study focusing on amenable mortality found differential funding increases to more deprived areas from 2001 to 2011 significantly contributed to inequalities narrowing,^23^ owing possibly to the way in which funds were targeted at primary care access, supply and quality in poorer areas.^24^

Since 2013, deprived areas in England have received proportionally less than previously.^25^ Furthermore, reduced overall NHS investment has delayed progress in redistributing funding to areas historically underfunded under the ‘pace of change’ policy.^26^ Given the significant changes in fiscal and health policy since 2010 and emerging indications that such reforms are damaging population health, we sought to repeat the previous analysis^23^ conducted by one of our authors, using secondary NHS data to explore whether changes in overall funding and the distribution of those funds in England between 2007 and 2014 are having an impact on the gap in rates of amenable mortality.

## Methods

### Setting and data sources

We undertook a time-series analysis using secondary data sources from 2007 to 2014 across 324 lower-tier local authorities in England, using 2009 boundaries and excluding the City of London and Isles of Scilly due to small population size.

For outcome data, we extracted age-standardised under 75-year male and female mortality rates from conditions amenable to healthcare from NHS Digital.^27^ Amenable mortality can assess health system performance and comprises mortality judged to constitute a failure to deliver quality or timely healthcare (see online supplementary appendix 1).^20 21^ NHS Digital also provided outcome data for rates of amenable mortality excluding ischaemic heart disease (IHD) and from causes considered not to be amenable to healthcare for model robustness analyses.

10.1136/jech-2018-211141.supp1 Supplementary data

Our main exposure variable was funding allocations to local NHS commissioners 2007–2014 which we collected from the department of health (see online supplementary appendix 2 for full detail). To create analytical consistency in time and place, we mapped allocations to local authorities using population counts at lower-layer super output area (LSOA) level, a statistical geography with population 1000–3000, and the development of a lookup between different commissioners and geographical levels (see online supplementary appendix 2). Allocations were inflation-adjusted using gross domestic product deflators for 2015/2016 and thereafter converted to per capita allocations using 2011 census populations.^28^

Previous related analyses found household income and unemployment rates were important confounders^22^; we therefore incorporated in our analysis annual gross disposable household income and unemployment benefit claimant rates from ONS.^29 30^

### Statistical methods

We first explored descriptive trends in NHS allocations and rates of amenable and non-amenable mortality, by quintile of deprivation, using the income component of the 2000 Index of Multiple Deprivation (IMD) to rank areas.^31^ This ensured a clear temporal baseline and allowed comparison to previous analyses.^22^ For local authorities merging since 2000, population-weighted average IMD scores were produced using population counts.^28^ We presented absolute and relative changes among the 20% most deprived and least deprived areas, based on accepted practice in reporting health inequalities^32 33^ and to maximise comparability with previous research.^22^

Thereafter, we developed a fixed-effects linear regression model using the PLM package in R (V.3.4.1)^34 35^ to estimate the effect of changes in NHS allocation on amenable mortality rates, controlling for household income and unemployment as confounders. We incorporated fixed-effects for local authorities and time trends to model unobserved heterogeneity and confounding.^36^ We explored variation in the impact of allocation on amenable mortality using an interaction term between funding and deprivation quintile, to understand the differential impact of funding on population inequalities. Finally, we fitted robust SEs to reflect probable spatial and serial autocorrelation.^37^ Models were estimated separately for male and female amenable mortality (see online supplementary appendix 3).

### Robustness tests

We performed standard regression diagnostics, testing for non-linearity between exposure and outcome variables, normality of residuals and homoscedasticity (see online supplementary appendix 4). Next, we tested our model specificity by exploring the relationship between funding and potential years of life lost (PYLL) due to mortality from amenable causes, amenable mortality rates excluding IHD and mortality from causes not amenable to healthcare (see online supplementary appendix 5). We posited that any association between funding and amenable mortality would be similarly identified with variables involving indices of mortality amenable to healthcare (amenable mortality excluding IHD and PYLL from amenable causes), but not for non-amenable mortality.

## Results

### Descriptive analysis

**Figure 1:**
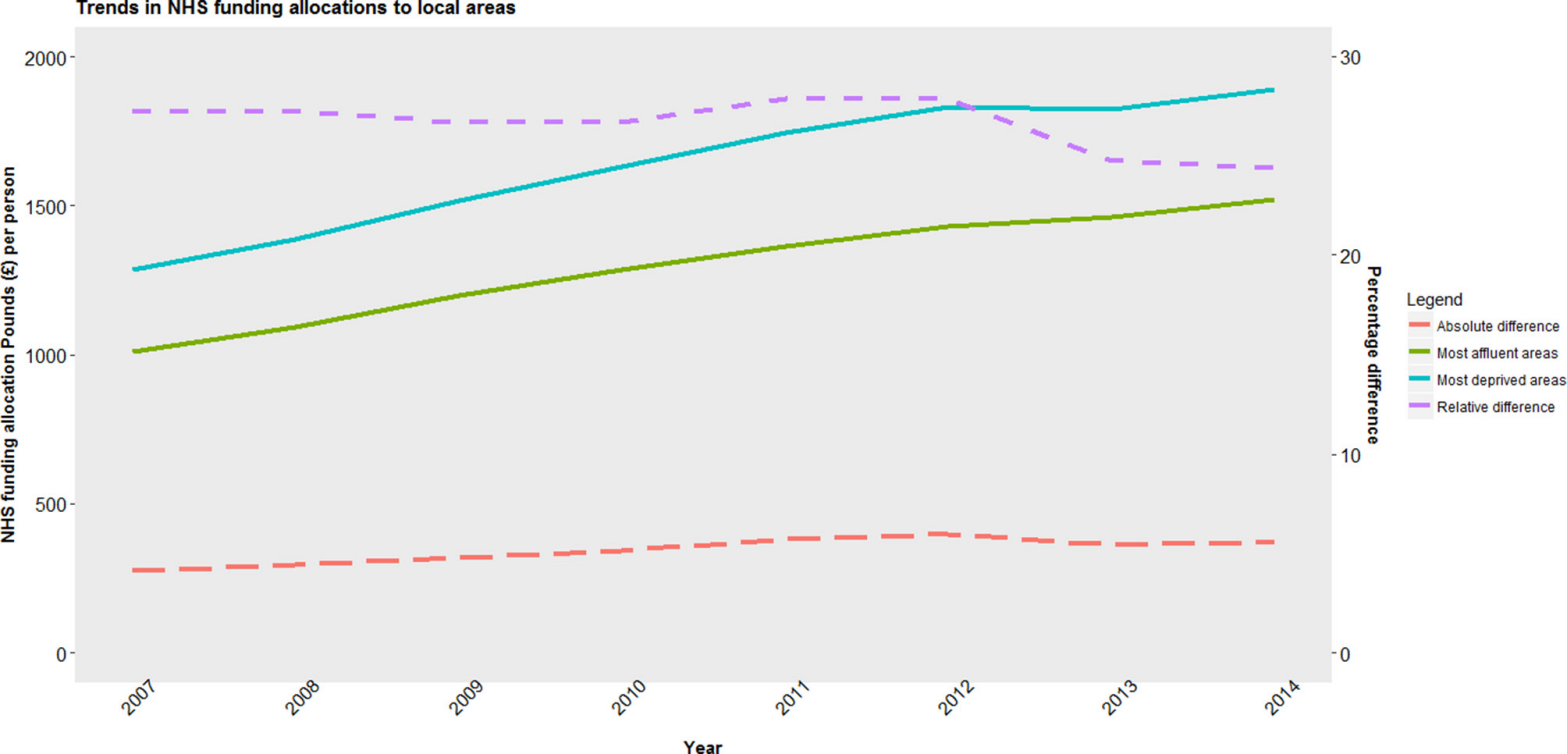
Trend in population-weighted average National Health Service (NHS) allocation per person to and inequalities in funding between most deprived and least deprived areas in England, 2007–2014.

NHS funding per head increased in real terms in quintile until 2012 (see figure 1). Between 2012 and 2013, the two most deprived quintiles’ allocations reduced, while the remaining three quintiles’ allocations rose. Funding increased per head from 2007 to 2014 by £499 in the most deprived quintile (37% increase), whereas the least deprived quintile received £471 per head more (45% increase).

**Figure 2:**
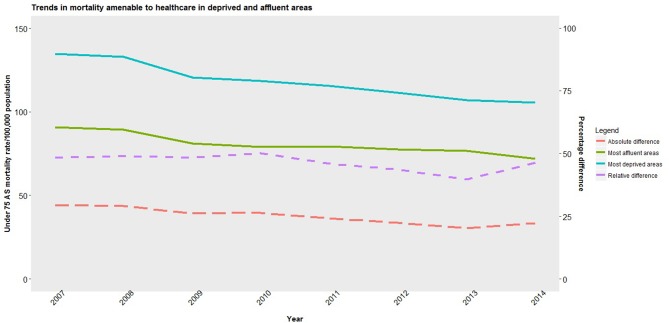
Trends in population-weighted average mortality amenable to healthcare for men in most deprived and least deprived areas and inequalities between areas in England, 2007–2014. AS, age standardised.

**Figure 3:**
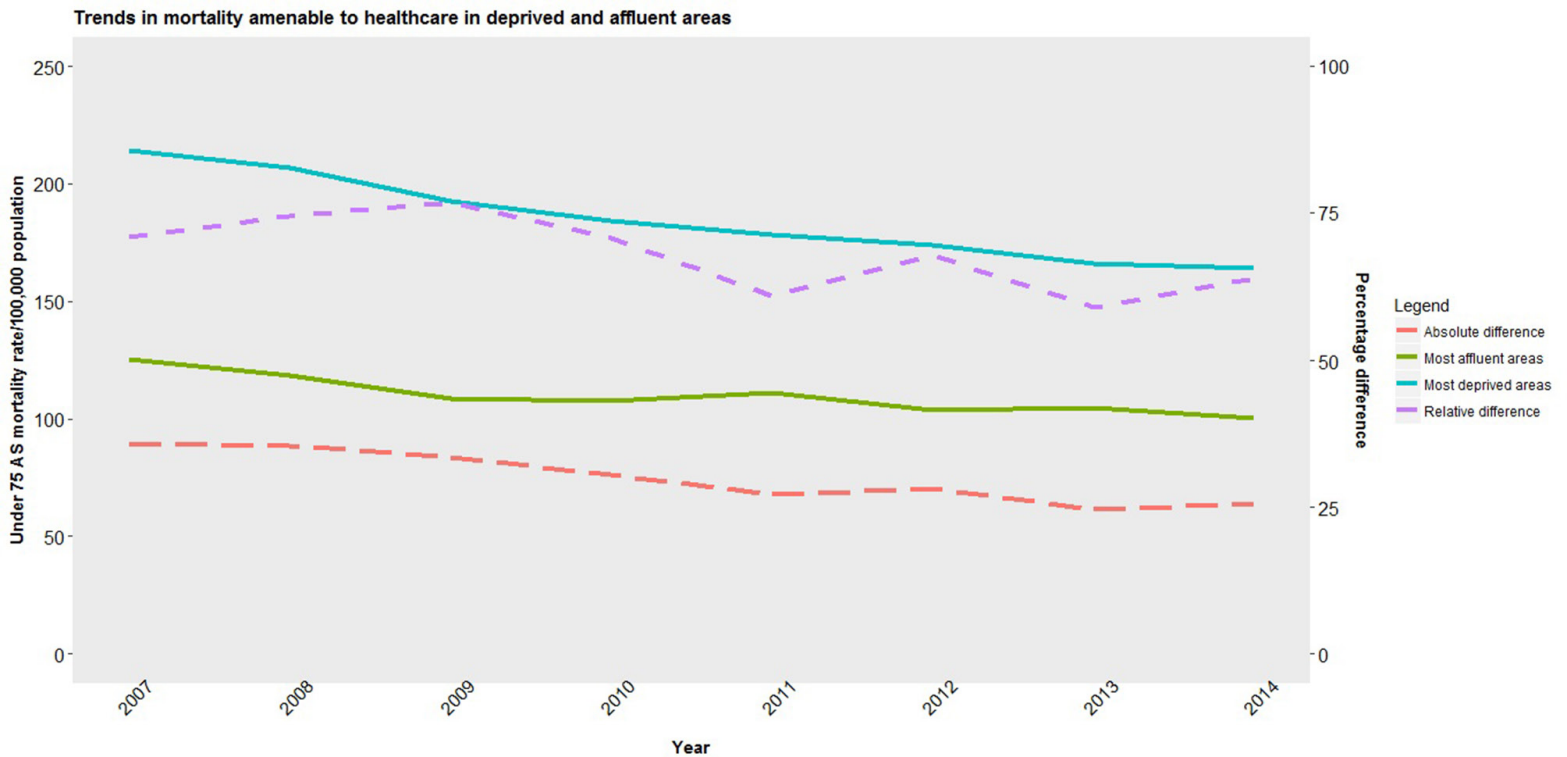
Trend in population-weighted average mortality amenable to healthcare for women in most deprived and least deprived areas and inequalities between areas in England, 2017–2014. AS, age standardised.

Male and female amenable mortality rates fell in 2007–2014, particularly in more deprived areas (figures 2 and 3). Male amenable mortality rates fell in the most deprived areas from 210 (95% CI 186 to 241) to 166 (144 to 190) per 100 000, a relative decline of 21.0% (20.96% to 21.4%), while in the least deprived areas they fell from 125 (94 to 156) to 102 (74 to 126) per 100 000 an 18.0% (17.93% to 18.07%) relative decline. Female amenable mortality rates fell in the most deprived areas from 133 (113 to 155) to 105 (89 to 126) per 100 000, a 21.0% (20.95% to 21.05%) relative decline, while in the least deprived areas they fell from 91 (65 to 116) to 72 (51 to 93) per 100 000, a 21.0% (20.92% to 21.08%) decline. Inequalities in male amenable mortality between the most and least deprived areas narrowed by 21 deaths per 100 000, a relative reduction of 67%– 62%. In contrast, though inequalities in female amenable mortality fell marginally by 9 deaths per 100 000 during the period, in relative terms inequalities rose from 45% to 46%. Levels of inequality in rates of male and female non-amenable mortality changed little (see online supplementary appendix 6). Finally, reductions in both amenable and non-amenable mortality appear since around 2009/2010 to be plateauing, particularly in deprived areas, while rates of non-amenable mortality in these areas appear to be increasing.

### Statistical analysis

Our regression analysis shows that increases in funding to the most deprived areas were associated with reductions in male amenable mortality, but not female (see table 1). Increases in funding of £500 per person were associated with a reduction in male amenable mortality of 13 deaths per 100 000 (95% CI −1.5 to −25; p=0.03). There was no significant association between funding changes in the most deprived areas and female amenable mortality, though it did approach significance (p=0.09).

**Table 1:** Changes in amenable mortality for men and women for every £500 additional per person investment in National Health Service (NHS) services, by local authority quintile of deprivation

Local authority deprivation quintile	Change in amenable mortality rate per 100 000 population for every £500 per head additional NHS funding allocation (95% CI)			
	Males	P values	Females	P values
First quintile (20% least deprived)	7.5 (−6.5 to 21.0)	0.30	3.0 (−7.5 to 13.0)	0.59
Second quintile	2.0 (−11.5 to 15.0)	0.79	1.5 (−7.5 to 10.0)	0.76
Third quintile	1.5 (−10.5 to 13.5)	0.80	−1.5 (−10.0 to 7.0)	0.72
Fourth quintile	−2.5 (−15.0 to 10.5)	0.72	−1.5 (−10.0 to 7.0)	0.72
Fifth quintile (20% most deprived)	−13.0 (−1.5 to 25.0)	0.03	−7.0 (−14.5 to 1.0)	0.09

95% CI based on robust SEs. Model based on equation 1 in online supplementary appendix 3. Model adjusted for local authority, annual trend, local unemployment rates and gross household income per head for each local authority.

Further analysis suggests NHS allocations accounted for a reduction in amenable mortality inequalities of 13 deaths per 100 000 population (1.5–25) for men (see online supplementary appendix 7 for calculations). The overall level of absolute inequality in male amenable mortality from 2007 to 2014 fell by 21 deaths per 100 000 suggesting that over half the reduction could be explained by differential allocations. Unlike previous research,^22^ trends in unemployment and household income were not as relevant in our model, though their inclusion did adjust coefficient estimates.

### Robustness tests

Diagnostics demonstrated the relationships between allocation and amenable mortality to be largely linear (see online supplementary appendix 3). Further checks for residual normality and homoscedasticity showed no violation of linear regression assumptions.

Specificity testing of our model revealed mixed findings: a similar association was found between allocation and PYLL to amenable causes as for amenable mortality (see online supplementary appendix 5); however, no significant association was found between funding and amenable mortality excluding IHD, while funding changes were associated changes in male non-amenable mortality, with increasing funding linked with greater mortality reductions, an association that grew in more disadvantaged areas.

### Discussion

Following a change of government in 2010 with a diminished focus on narrowing inequalities, our study has shown that inequalities in male and female amenable mortality continued to fall in absolute terms from 2007 to 2014. The reduction in inequality among men appears to be explained by differential funding to more deprived areas under NHS resource allocation policy. In comparison, our model did not find an association between funding and reductions in inequalities in female amenable mortality, though this may have been due to a lack of power. Of perhaps greatest concern however, amenable mortality rates in the most deprived areas since 2009/2010 appear to be worsening, a trend which should it continue may erode past gains.

### Comparison with other studies

Our findings corroborate previous research showing changes in health policy can impact on inequalities in amenable mortality.^23 38 39^ Our study showed inequalities in amenable mortality fell between 2007 and 2014 in both absolute and relative for males, and in absolute terms for females. One study by Asaria *et al*^24^ found relative inequalities between 2004 and 2012 to rise for both genders while absolute inequalities fell. These authors however aggregated individual mortality at an LSOA level, not local authority, while no attempt was made to adjust for socioeconomic confounders.

Our analysis also suggests a more conservative relationship between funding and reductions in amenable mortality than in previous studies in England.^22^ Reductions in amenable mortality may have become more refractory to funding increases over time. Alternatively, previous analyses took place during record investment in the NHS and a policy focus on tackling health inequalities; diminished funding and focus since 2010 in addition to the national reconfiguration of the health service may have distracted from this agenda.

The association between allocation and alternative mortality measures diverged in our model from that of other studies^23^ which have shown a more consistent relationship between allocation and inequalities in amenable mortality. Given excluding deaths from IHD in our analysis attenuated the relationship, and non-amenable mortality includes 50% of IHD deaths, it is possible that cardiovascular deaths may have been responsible for the association; debate continues on the avoidability of IHD deaths and their classification as amenable^40–42^ Alternatively, some unobserved confounder may have influenced the amenable and non-amenable mortality trends observed.

### Strengths and limitations

Our analytical approach has, we believe, several strengths. Our longitudinal approach increased the possibility that our exposure and outcome variables are causally linked. Second, unlike other studies,^24^ we controlled for recognised socioeconomic confounders. Third, we applied a granular approach to mapping local NHS allocations to local authorities, using LSOA populations. Finally, we adjusted our findings using robust SEs, reflecting inevitable spatial and serial autocorrelation.

However, our findings should be considered in light of some limitations. There is likely to be multicollinearity in our model between IMD and socioeconomic confounders, inflating SEs of coefficients and biasing our results towards the null hypothesis. As shown above, changes in funding were associated with a non-equivalent variable (non-amenable mortality) and not with one of the equivalent variables (amenable mortality excluding IHD). Our study also employed a somewhat narrow focus on amenable mortality in people aged under 75 years, with a particular concentration on more deprived groups: budgetary reductions may be causing wider challenges to other social groups and services. Finally, we cannot ignore the risk that unaccounted confounding variables were responsible for the changes in outcome witnessed.

### Implications for future research and policy

Our study’s findings make a number of compelling claims for policy-makers. First, despite a short period since the change in government and funding, trends in inequalities in amenable mortality appear far less favourable than in previous years. Our analysis was challenged by the reconfiguration of healthcare commissioning in England in 2013; to our knowledge, no analysis by the department of health has compared local allocations before and after the reforms to demonstrate equity in the transfer of commissioning. Our findings suggest more deprived areas suffered the most from the reconfiguration, and require further research to evaluate.

Second, our findings confirm that health services have a role in addressing health inequalities: deaths from amenable causes comprise approximately one-third of total deaths, making it a ripe target for improvement. Downward revisions to deprivation weighting in funding formulae, combined with limited overall investment and consequent slowing of progress towards target allocations will have significant effects. This lost opportunity of what is arguably a far less intractable source of mortality inequalities should we believe make healthcare planners take stock and reconsider their efforts to exploit the NHS’s role in tackling inequalities.

Finally, though post-hoc analysis of England’s Health Inequalities Strategy demonstrated it to have been clearly successful,^18^ it would appear since the abandonment of this policy focus that progress is being reversed. Reductions in amenable mortality have plateaued, while rates of remaining causes of mortality in deprived areas appear to be rising. While the Health and Social Care Act^43^ compels ministers and commissioners to act on inequalities, gone is the national strategy and cross-departmental collaboration that was previously witnessed.^44^ Instead, a focus on balanced budgets and marginal increases or cuts to budgets have become the new focus, at the expense of health equity. Given the impact of health inequalities to social cohesion,^45^ economic productivity^46^ and government spending,^47^ the country cannot afford not to address this. A fresh national strategy to tackle inequalities learning from previous years and renewed investment could we believe achieve this in a short space of time.

## Conclusions

Compared with previous periods, 2007–2014 saw proportionally less healthcare funding to deprived areas while overall increases in health spending slowed. Differential investment in deprived areas contributed to a reduction in amenable mortality for men, though not for women. With diminished political focus on narrowing inequalities since 2010, careful scrutiny is needed to ensure previous gains are not being lost.

**What is already known on this subject**

- Rates of mortality in England due to conditions potentially amenable to healthcare vary significantly between socioeconomic groups with more deprived areas displaying far higher rates.
- Previous analyses of policies aiming to address mortality inequalities have shown apportioning greater levels of healthcare funds to more deprived areas to be one effective approach of tackling such inequalities between more deprived and affluent areas.

**What this study adds**

- Despite lower funding since 2010, a financial recession in 2008 and a reorganisation of the National Health Service (NHS) in England in 2013, inequalities in amenable mortality continued to fall between 2007 and 2014 for men and women.
- Data from more recent years however suggest successive gains are slowing and may more worryingly be in reverse.
- Our model demonstrates differential NHS investment in more deprived areas to remain to be a cost-effective and potent way of addressing health inequalities alongside wider more upstream strategies.
