# Supplementary material for: Evaluating effects of recent changes in NHS resource allocation policy on inequalities in amenable mortality in England, 2007–2014: time-series analysis
Source: J Epidemiol Community Health. 2018 Nov 23;73(2):162–7. doi: 10.1136/jech-2018-211141 (PMC6352397; doi:10.1136/jech-2018-211141)
Supplement: Supplementary data [file jech-2018-211141supp001.pdf]

Supplementary appendix 1: Causes of death considered to be amenable to healthcare

NHS Digital defines deaths considered amenable to healthcare if it is considered they could be averted if timely, appropriate and high-quality healthcare could prevent their occurrence.[4,8] Deaths may be specific to an age group and are classified by underlying cause of death using the ICD-10 classification, registered in the relevant calendar year and are aggregated to population level.

**Causes of death considered amenable to healthcare**		
**Name of group**	**Age**	**ICD-10 code**
Intestinal infections[a]	0-14	A00-A09
Tuberculosis	0-74	A15-A19, B90
Other infections (Diphtheria, Tetanus, Poliomyelitis)[a]	0-74	A36, A35, A80
Whooping cough[a]	0-74	A37
Septicaemia[a]	0-74	A40-A41
Measles[a]	1-14	B05
Selected invasive bacterial & protozoal infections)[b]	0-74	A38-A41, A46, B50-B54, G00, G03, J02, L03
Hepatitis C[b]	0-74	B17.1, B18.2
HIV/AIDS[b]	0-74	B20-B24
Malignant neoplasm of colon and rectum	0-74	C18-C21
Malignant neoplasm of skin	0-74	C44
Malignant neoplasm of breast	0-74	C50
Malignant neoplasm of cervix uteri	0-74	C53
Malignant neoplasm of cervix uteri and body of the uterus[a]	0-74	C54, C55
Malignant neoplasm of testis[a]	0-74	C62
Hodgkin's disease	0-74	C81
Leukaemia	0-74	C91-C95[a]<br>C91, C92.0[b]
Benign neoplasms[b]	0-74	D10-D36
Diseases of the thyroid[a]	0-74	E00-E07
Diabetes mellitus	0-49	E10-E14
Epilepsy	0-74	G40-G41
Rheumatic and other valvular heart disease	0-74	I05-I09[a]<br>I01-I09[b]
Hypertensive disease	0-74	I10-I13, I15[a]<br>I10-I15[b]
Ischaemic heart disease	0-74	I20-I25
Cerebrovascular disease	0-74	I60-I69
All respiratory diseases (excl. pneumonia/influenza)[a]	1-14	J00-J09, J20-J99
Influenza	0-74	J10-J11[a]<br>J09-J11[b]
Pneumonia	0-74	J12-J18
Asthma	0-44[a]<br>0-74[b]	J45-J46
Peptic and duodenal ulcers	0-74	K25-K27[a]<br>K25-K28[b]
Appendicitis[a]	0-74	K35-K38
Abdominal hernia[a]	0-74	K40-K46
Cholelithiasis & cholecystitis[a]	0-74	K80-K81

Acute abdomen, appendicitis, intestinal obstruction, cholecystitis/lithiasis, pancreatitis, hernia[b]	0-74	K35-K38, K40-K46, K80-K83, K85, K86.1-K86.9, K91.5
Nephritis and nephrosis	0-74	N00-N07, N17-N19, N25-N27
Benign prostatic hyperplasia[a]	0-74	N40
Obstructive uropathy & prostatic hyperplasia	0-74	N13, N20-N21, N35, N40, N99.1
Maternal deaths[a]	All	O00-O99
Congenital cardiovascular anomalies[a]	0-74	Q20-Q28
Perinatal deaths, all causes excluding stillbirths[a]	All	A34[a]<br>P00-P96, A33
Congenital malformations, deformations & chromosomal abnormalities[b]	0-74	Q00-Q99
Misadventures to patients during surgical and medical care	All	^60-Y69, Y83-Y84

Source: HSCIC, 2015[1]

[a] classified up to 2010

[b] classified from 2011

Supplementary appendix 2: Method of generating annual figures for funding data for NHS spending in England between 2007-2014 for comparable areas

Responsibility for health commissioning altered in 2012/13 in England, dividing responsibilities previously held by Primary Care Trusts (PCTs) to Clinical Commissioning Groups for secondary care, Local Area Teams (LATs) for primary care, local authorities for public health and NHS England for specialised services.

We extracted figures for budget allocations for PCTs from 2007-2013[2] and from 2013-2014 from CCGs for secondary care, [3] LATs for primary care,[4] local authorities for public health[5] and figures for specialised budget allocations mapped to CCG level.[6]

We then converted these funding figures to lower-tier local authority areas by developing a lookup between each geographical level using existing published lookups from the Office for National Statistics[7,8,9,10,11,] calculating figures at a Lower Layer Super Output Area level and aggregating these to local authorities using published population counts.

Supplementary appendix 3: Regression Model Formula

Model equation:

$Mortality_{i,j} = \beta_1 Allocation_{i,I} + \beta_2 GDHI_{i,j} + \beta_3 Unemployment_{i,j} + IMDQ_j \text{ x } Allocation_{i,j} + \mu_j + t + \varepsilon_{i,j}$

Where i is the year of analysis and j is the local authority. Allocation, GDHI and Unemployment are the NHS funding allocation, average gross disposable household income and unemployment rate in, local authority j and year i. t and μ are the fixed-effects variables for time and local authorities while ε is the error term. A series of interaction terms have been introduced to explore the relationship with funding and mortality in each quintile of deprivation ($IMDQ_i$).

Supplementary appendix 4: Regression Diagnostics

1. **Confirming normality assumption**

The assumption that our regression model's residuals were normally distributed was tested by plotting quantiles of rediduals against quantiles of the normal distribution. The approximation to the fitted line suggests the residuals are approximately normally distributed.

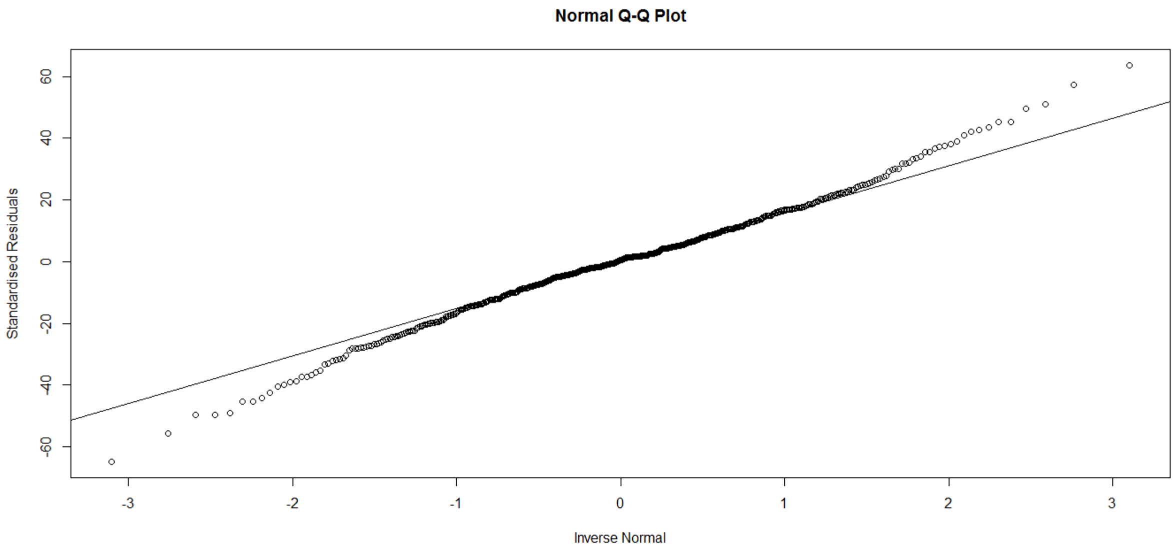

Figure 1 – Quantiles of model residuals against quantiles of the normal distribution

This is confirmed when we plot a histogram our model residuals in a histogram with the normal distribution fitted.

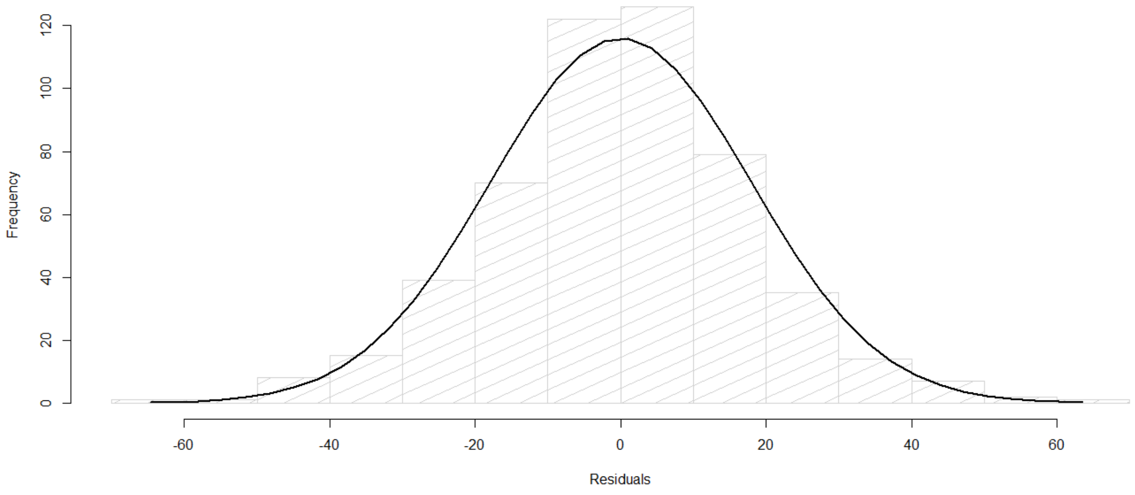

Figure 2 – Histogram of model residuals

2. **Testing for presence of heteroscedasticity**

Next, we tested whether the standard deviations of the error terms were correlated with the predictor variables in our model. The uniform spread of points in the y-direction on moving along

the x-axis suggests constant variance in the residuals, suggesting there is limited heteroscedasticity in our model.

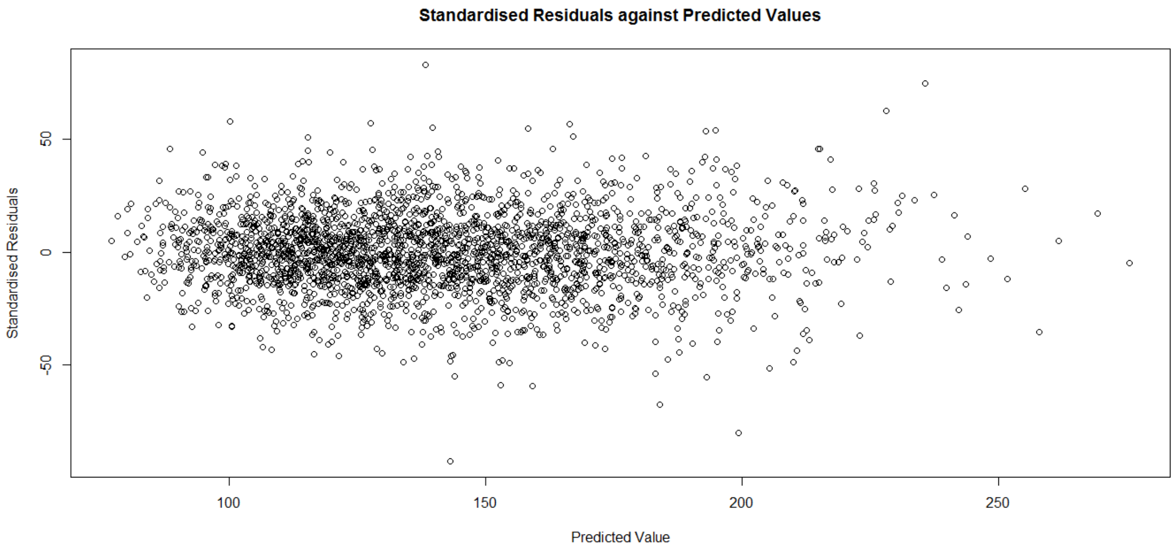

Figure 3 – Plot of model residuals against fitted values of predictor variables

3. Testing for presence of non-linearity between predictor and outcome variables

Lastly, a component plus residual plot was constructed to explore the presence of linearity between the main predictor variable, allocation, and the response variable, taking into account the presence of other independent variables. The regression line below confirms a linear relationship through the scatterplot, indicating that the assumption of a linear relationship to be reasonable.

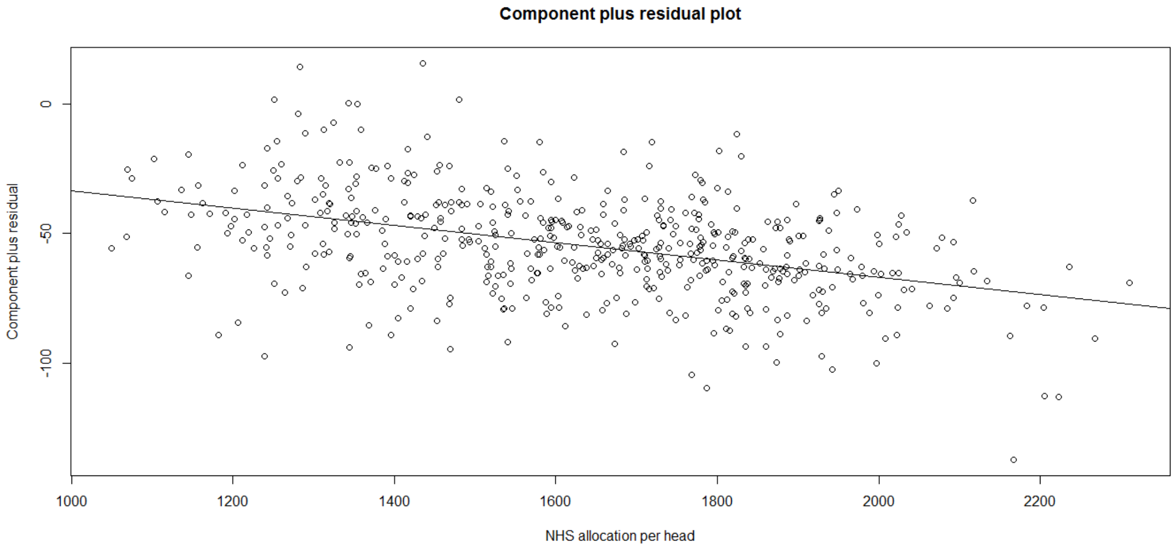

4. Testing for multicollinearity using a pooled model

Variable	Generalised Variance Inflation Factor (GVIF)	Degrees freedom	$GVIF^{1/(2 \times DF)}$
Allocation	8.83	1	2.97
GDHI	1.33	1	1.15
Unemp	2.31	1	1.52
IMDQ	4.63	4	1.21
Allocation*IMDQ	12.63	4	1.31

Variance inflation factors of over 5 generally represent potential for multicollinearity, while figures exceeding 10 are signs of significant multicollinearity. The GVIF for the interaction variable between allocation and IMDQ exceeds 10 and therefore highlights the likely presence of significant multicollinearity in the pooled model.

Supplementary appendix 5: Alternative model output

Allocation and PYLL

	Male	*LCI*	*UCI*	*Female*$^{-}$	*LCI*	*UCI*
IMDQ1	0.116	-0.542	0.775	0.024	-0.565	0.612
IMDQ2	-0.036	-0.669	0.598	-0.041	-0.607	0.525
IMDQ3	-0.019	-0.618	0.579	-0.091	-0.644	0.463
IMDQ4	-0.296	-0.919	0.327	-0.013	-0.563	0.537
IMDQ5	-0.644	-1.214	-0.074	-0.337	-0.832	0.158

Allocation and AM excluding IHD

	Male	*LCI*	*UCI*	*Female*$^{-}$	*LCI*	*UCI*
IMDQ1	0.017	-0.002	0.036	0.001	-0.017	0.019
IMDQ2	-0.002	-0.020	0.017	-0.001	-0.017	0.016
IMDQ3	0.011	-0.007	0.029	-0.020	-0.017	0.013
IMDQ4	0.005	-0.012	0.022	-0.003	-0.018	0.013
IMDQ5	-0.001	-0.017	0.017	-0.005	-0.018	0.009

Allocation and non-AM

	Male	*LCI*	*UCI*	*Female*$^{-}$	*LCI*	*UCI*
IMDQ1	-0.008	-0.047	0.031	0.017	-0.013	0.046
IMDQ2	-0.009	-0.044	0.025	0.015	-0.014	0.044
IMDQ3	-0.020	-0.055	0.014	0.018	-0.009	0.046
IMDQ4	-0.022	-0.057	0.013	0.020	-0.006	0.046
IMDQ5	-0.048	-0.079	-0.017	0.005	-0.020	0.030

Supplementary appendix 6: Trends in mortality due to causes not amenable to healthcare for men and women between 2007 and 2014

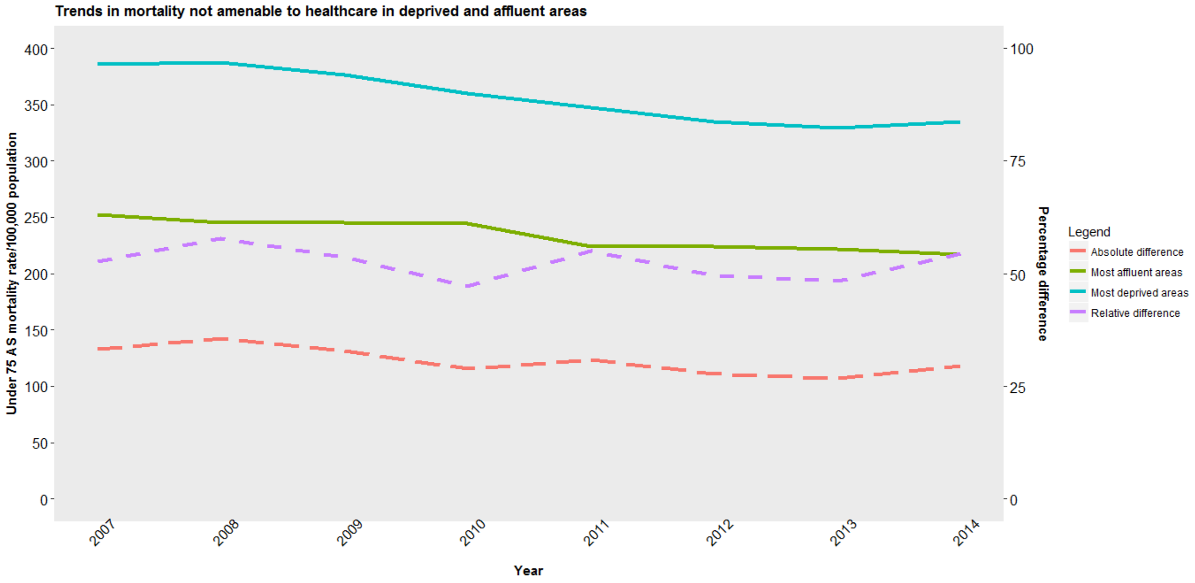

Figure 1 - Trend in population-weighted average mortality not amenable to healthcare for men in deprived and affluent areas and inequalities between areas in England, 2017 to 2014. AS=age standardised.

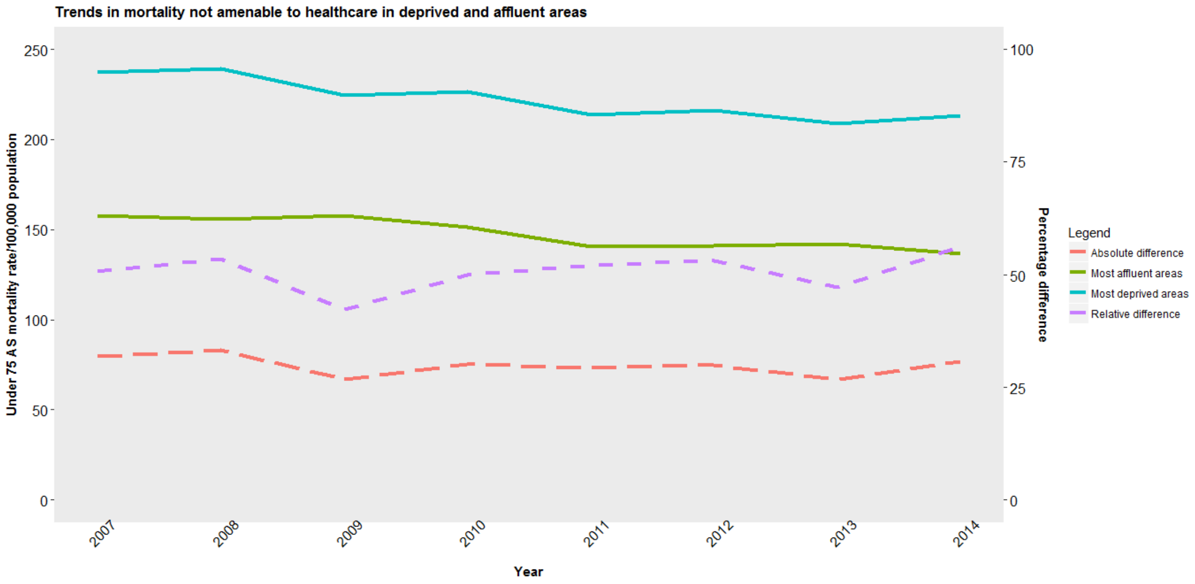

Figure 2 - Trend in population-weighted average mortality not amenable to healthcare for women in deprived and affluent areas and inequalities between areas in England, 2017 to 2014. AS=age standardised.

Supplementary appendix 7: Calculating the reduction in mortality amenable to healthcare in deprived areas from regression estimates

Our model estimates the mortality reduction per pound of additional per person NHS spending between 2007 and 2014. For intuitiveness the figures displayed in Table 1 detail mortality reductions associated with greater changes in spending to the level of an additional £500 per person at a local level. Our model estimated each additional £1 per person NHS funding was associated in our model in deprived areas with a reduction of 0.026 (95% confidence interval 0.003 to 0.050) male deaths per 100,000 population. There was no significant association in the most affluent areas. NHS funding in the most deprived areas increased by £499 per person; using our model estimates this would theoretically produce a reduction of 13 deaths:

= 499 * 0.026 = 13

UCL = 499 * 0.050 = 25

LCL = 499 * 0.003 = 1.5

---

[1] Health & Social Care Information Centre (HSCIC). Compendium of Population Health Indicators. Mortality from causes considered amenable to health care. Indicator specification document. December 2015. Available from: https://indicators.hscic.gov.uk/download/NCHOD/Specification/Spec_03D_171DRT0074.pdf [Accessed 3 July 2017].

[2] Department of Health. NHS Allocations. Exposition books. Available from: http://webarchive.nationalarchives.gov.uk/+/http://www.dh.gov.uk/en/Managingyourorganisation/Financeandplanning/Allocations/index.htm [Accessed 27 Feb 2018].

[3] NHS England. Allocations. Allocations for 2013/2014. Available from: https://www.england.nhs.uk/allocations [Accessed 27 Feb 2018].

[4] NHS England. Technical Guide to Clinical Commissioning Group and Area Team allocations 2014-15 and 2015-16. K – Primary Care. Available from: https://www.england.nhs.uk/2014/03/allocations-tech-guide [Accessed 27 February 2018].

[5] Department of Health and Social Care. Public health grants to local authorities from 2013 to 2016. Available from: https://www.gov.uk/government/publications/ring-fenced-public-health-grants-to-local-authorities-2013-14-and-2014-15 [Accessed 27 Feb 2018].

[6] NHS England. Annex D: Specialised Services. Available from: https://www.england.nhs.uk/publication/annex-d-specialised-services [Accessed 27 February 2018].

[7] Output Area to Primary Care Organisation to Strategic Health Authority (December 2011) Lookup in England and Wales. Available from: https://data.gov.uk/dataset/output-area-to-primary-care-organisation-to-strategic-health-authority-december-2011-lookup-in-4 [Accessed 27 February 2018].

[8] ONS. Lower Layer Super Output Area (2011) to Ward (2016) Lookup in England and Wales. Available from: http://geoportal.statistics.gov.uk/datasets/lower-layer-super-output-area-2011-to-ward-2016-lookup-in-england-and-wales [Accessed 27 February 2018].

[9] ONS. LSOA (2011) to Clinical Commissioning Groups to Sustainability and Transformation Partnerships (April 2017) Lookup in England. Available from: http://geoportal.statistics.gov.uk/datasets/lsoa-2011-to-clinical-commissioning-groups-to-sustainability-and-transformation-partnerships-april-2017-lookup-in-england [Accessed 27 February 2018].

[10] NHS Commissioning Board: Local area teams. Available from: https://www.england.nhs.uk/2012/06/local-teams-senates [Accessed 27 February 2018].

[11] ONS. Lower Tier Local Authority to Upper Tier Local Authority (December 2017) Lookup in England and Wales. Available from: http://geoportal.statistics.gov.uk/datasets/lower-tier-local-authority-to-upper-tier-local-authority-december-2017-lookup-in-england-and-wales [Accessed 27 February 2018].
